# Understanding the Protective Role of Exosomes in Doxorubicin-Induced Cardiotoxicity

**DOI:** 10.1155/2022/2852251

**Published:** 2022-09-12

**Authors:** Guoxia Zhang, Xinyu Yang, Xin Su, Na An, Fan Yang, Xinye Li, Yuchen Jiang, Yanwei Xing

**Affiliations:** ^1^Guang'anmen Hospital, China Academy of Chinese Medical Sciences, Beijing, China; ^2^Fangshan Hospital, Beijing University of Chinese Medicine, Beijing 100700, China; ^3^Beijing University of Chinese Medicine, Beijing, China

## Abstract

Doxorubicin (DOX) is a class of effective chemotherapeutic agents widely used in clinical practice. However, its use has been limited by cardiotoxicity. The mechanism of DOX-induced cardiotoxicity (DIC) is complex, involving oxidative stress, Ca^2+^ overload, inflammation, pyroptosis, ferroptosis, apoptosis, senescence, etc. Exosomes (EXOs), as extracellular vesicles (EVs), play an important role in the material exchange and signal transmission between cells by carrying components such as proteins and RNAs. More recently, there has been a growing number of publications focusing on the protective effect of EXOs on DIC. Here, this review summarized the main mechanisms of DIC, discussed the mechanism of EXOs in the treatment of DIC, and further explored the value of EXOs as diagnostic biomarkers and therapeutic strategies for DIC.

## 1. Introduction

The survival duration of cancer patients has increased as medical technology has advanced, yet cardiovascular toxicity has emerged as one of the most significant side effects of cancer treatment [1]. Cancer survivors are eight times more likely than the normal population to suffer cardiovascular disease [2]. DOX is one of the most effective chemotherapeutic agents used in clinical practice, with indications for a wide range of cancers. Nevertheless, its usage has been limited due to the high risk of cardiotoxicity [3], which may be acute, early, or late, including pericarditis, heart failure, and arrhythmia. Studies have shown that the incidence of DOX-induced left ventricular dysfunction ranges from 3% to 48% in a dose-dependent manner [2, 4–6]. Therefore, fully understanding the mechanism of DIC, conducting effective monitoring at an early stage, and taking effective prevention and intervention strategies are the key issues to improve the quality of life of cancer patients.

However, the pathogenesis of DIC is complex, involving oxidative stress, inflammation, Ca^2+^ dysregulation, senescence, apoptosis, pyroptosis, ferroptosis, etc. [7–9] Besides, there is currently a lack of biomarkers for early diagnosis of DIC that are both specific and sensitive [10]. Although dexrazoxane is the only cardioprotective agent recommended by the FDA to reduce DIC, its application is only for adults with advanced or metastatic breast cancer who have received a cumulative dose of >300 mg/m^2^ DOX [4, 11], and its protective effect has been questioned in some studies [12].

EXOs are EVs with a diameter of 50 nm to 150 nm formed by cells through a series of regulatory processes (endocytosis, fusion, and excretion), which play an important role in cell communication and tissue microenvironment regulation [13]. Increasing evidence suggests that some components in EXOs, such as miRNAs, lncRNAs, and proteins, are transferred into cardiomyocytes and exert cardioprotective effects through different signaling pathways [14–16].

In this review, we outline the evidence on EXOs' mechanisms for mitigating DIC. As well, we deliver an overview regarding the diagnostic biomarkers and therapeutic effects of EXOs, hoping to provide a theoretical basis for the clinical application of EXOs.

## 2. Introduction to EXOs and EVs

EVs are membrane-derived vesicles ranging from 50 nm to 2,000 nm in diameter released by cells into the extracellular space [17]. All different types of cells in mammals including neuronal cells, endothelial cells (ECs), mesenchymal stem cells (MSCs), and epithelial cells can release EVs, and EVs are widely distributed in the body and can be detected in urine, blood, saliva, and other body fluids [18]. EXOs are EVs with a size range of ~40 to 160 nm (average ~100 nm) in diameter with an endosomal origin. Depending on the cell of origin, EVs, including EXOs, can contain many constituents of a cell, including DNA, RNA, lipids, metabolites, and cytosolic and cell surface proteins [19].

**Figure 1:**
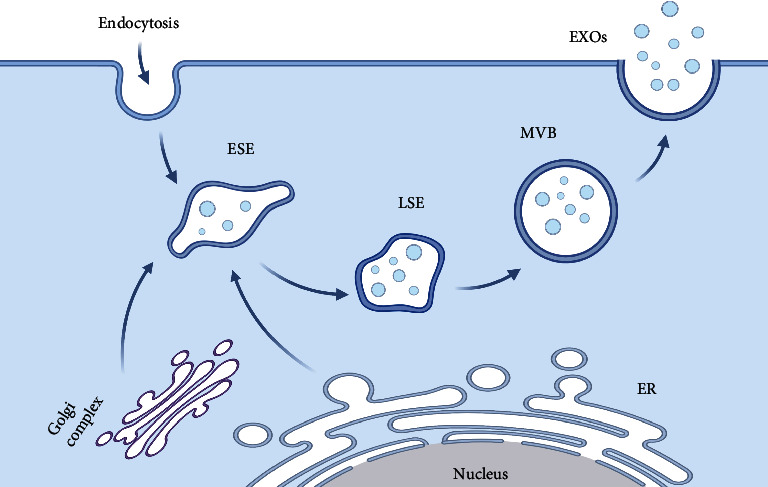
The biosynthesis of EXOs. ESE: early-sorting endosome; LSE: late-sorting endosome; MVB: multivesicular body; EXOs: exosomes; ER: endoplasmic reticulum.

Generally speaking, EXOs originate from the endosomal pathway. First, the cytoplasmic membrane invaginates to form early-sorting endosomes (ESEs) containing some extracellular components and membrane surface proteins. The contents of the ESEs are obtained by fusing with the previously existing ESEs or exchanging substances with other organelles. ESEs then develop further within the cell into late-sorting endosomes (LSEs). LSEs can eventually form multivesicular bodies (MVBs), which contain intraluminal vesicles (ILVs) that are formed by budding from the inward depression of the multivesicular body membrane. The fusion of MVBs and the cytoplasmic membrane leads to the secretion of ILVs outside the cell, and these ILVs are EXOs [20] (Figure 1).

EXOs have different sizes, contents, and origins, leading to complex heterogeneity. EXOs are rich in proteins, lipids, miRNAs, and other substances, and the contents enriched in different EXOs are different [21]. Differences in EXO composition, especially in cell surface proteins, can have different effects on recipient cells. EXOs can mediate intercellular communication under physiological and pathological conditions [22]. Parental cells that released EXOs are absorbed by receptor cells, through the exchange of substances or release of inclusions to achieve the exchange of substances and signals, to play an important role in reproductive development, immune response, disease occurrence, and other biological processes [18, 19, 21, 23]. Studies have shown that EXOs are closely related to cardiovascular diseases, neurodegenerative diseases, tumor growth and metastasis, and drug resistance [19, 24, 25].

## 3. The Mechanism of DIC

**Figure 2:**
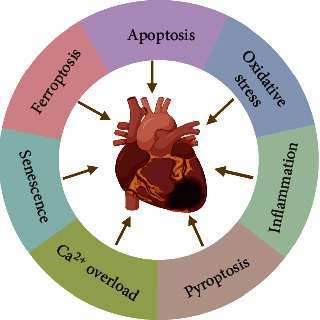
The main mechanisms of DIC.

The mechanism of DIC is complex, and there is no clear mechanism to explain it. Multiple pathways may be involved, including oxidative stress, apoptosis, autophagy, pyroptosis, ferroptosis, and senescence, which are thought to be interconnected and act together to cause myocardial damage [26]. Herein, we have summarized molecular mechanisms which are involved in DIC briefly (Figure 2).

### 3.1. Oxidative Stress

The oxidative stress hypothesis involving the intramyocardial production of reactive oxygen species (ROS) has gained the most widespread acceptance [27, 28]. Generally speaking, under the action of nitric oxide synthase (NOS) and NADPH oxidase (NOX), DOX is reduced to semiquinone DOX, which reduces oxygen to superoxide anion; this radical is converted into hydrogen peroxide under the action of superoxide dismutase (SOD). Hydrogen peroxide can be cleaved into hydroxyl radicals [28–32]. The above-mentioned ROS, especially hydroxyl radicals, are active and highly toxic, which can cause lipid peroxidation, thereby destroying the biofilm structure and causing myocardial cell damage and death.

### 3.2. Apoptosis

The role of apoptosis in DIC is well-established. Many studies have shown that DOX can activate cardiomyocytes to undergo apoptosis. DOX upregulated the expression of heat shock protein 25 (HSP25), which transactivated p53, leading to the expression of Bax, therefore inducing the apoptotic death of cardiomyocytes [33]. DOX also activated caspase-3 [34, 35]. Besides, DOX can induce cardiomyocyte apoptosis by activating the nuclear factor kappa-B (NF-*κ*B) signaling pathway and producing ROS [36–38]. DOX significantly upregulated the expression of death receptors (DRs) (TNFR1, Fas, DR4, and DR5) and subsequently induced apoptosis in iPS-derived cardiomyocytes, and the apoptosis could be accelerated by physiologically relevant death ligands including TNF-related apoptosis inducing ligand (TRAIL) [39]. DOX also downregulated caspase recruitment domain ARC, leading to Bax translocated from the cytosol to mitochondria, resulting in loss of mitochondrial membrane potential, which led to cytochrome C release, thus inducing apoptosis [40].

### 3.3. Inflammation

Inflammation also plays a role in DOX-triggered cardiac injury. DOX can increase the levels of proinflammatory cytokines such as interleukin- (IL-) 1*β*, IL-18, IL-6, and tumor necrosis factor-alpha (TNF-*α*) [41, 42], and their elevations are associated with the activation of the p38/MAPK/NF-*κ*B pathway [41, 43]. Besides, improving nuclear factor erythroid2-related factor 2 (Nrf2) signaling protected the heart from NF-*κ*B-mediated inflammatory injury [43]. One study suggested that DOX could cause upregulation of the proinflammatory toll-like receptor 4 (TLR4) in macrophages and endotoxin leaking from gut flora into the circulation, which combined to cause a systemic inflammatory response [44]. In addition, DOX can cause inflammation by activating the sirtuin 1-NOD-like receptor protein 3 pathway [45].

### 3.4. Pyroptosis

Pyroptosis in DIC is evidenced by increased cell death, upregulated expression levels of NLR family pyrin domain containing 3 (NLRP3), caspase-3, IL-1*β*, IL-18, and GMDSD-N, and morphological features [46–50]. Mechanistically, DOX upregulated the lncRNA TINCR, which can recruit IGF2BP1 to upregulate the expression of NLRP3 to induce pyroptosis [46]. In addition, by upregulating BH3-only protein Bcl-2/adenovirus E1B 19-kDa-interacting protein 3 (Bnip3), DOX induced the activation of caspase-3, causing GSDME-dependent pyroptosis [48].

### 3.5. Senescence

In recent years, many studies have demonstrated that low doses of DOX (≤0.5 *μ*M) preferentially induce cardiovascular cell senescence, rather than apoptosis [51–53]. In general, the mechanism of DOX-induced cardiovascular cell senescence involves oxidative stress, telomere damage, DNA damage, etc. In rat neonatal cardiomyocytes, low levels of DOX can induce senescence through oxidative stress [54]. It can also lead to telomere damage through p38-mediated reduction of telomere binding factors 2 (TRF2) and JNK/p53-mediated reduction of telomere binding factors 1 (TRF1), thereby inducing senescence [55]. 0.25, 0.5, and 1 *μ*M DOX can induce human primary vascular smooth muscle cell senescence [53, 56, 57]. Mechanistically, DOX upregulated uPAR, which led to TRF2 ubiquitination and proteasomal degradation, leading to senescence [56] and DOX-mediated elevation of ROS also aggravated senescence [57]. In addition, 0.25 *μ*M DOX activated MAPK-p38 to induce p16 (INK4A) expression and cytoskeleton remodeling, therefore inducing senescence [58]. In ECs, DOX induced senescence by upregulating p53-dependent XIAP-associating factor 1 expression [59]. In addition to the above in vitro and in vivo models, studies have shown that human cardiac progenitor cells also exhibited aging characteristics in DIC patients [60].

### 3.6. Ferroptosis

Ferroptosis is a new form of cell death proposed by Stockwell in 2012 [61]. Many scholars have found that DOX can induce ferroptosis in cardiomyocytes [47, 62]. Preadministration of ferroptosis inhibitors largely prevents DIC [62], whereas a high-iron diet can exacerbate DIC [63]. In general, the mechanism of DOX-induced ferroptosis can be divided into two aspects: induction of iron overload in cardiomyocytes and lipid peroxidation. Specifically, DOX can induce iron overload in cardiomyocytes by upregulating the level of transferrin receptor [64, 65] and heme oxygenase 1-mediated heme degradation [62]. DOX can also regulate iron metabolism-related genes by acting on iron-responsive elements/iron-responsive proteins [66–68]. Besides, the quinine moiety of DOX accepted electrons from NOX and NOS to become semiquinone, which was accompanied by the production of ROS [29], and DOX can inhibit the activity of intracellular glutathione peroxidase 4 (GPX4), reducing its antioxidant capacity [69, 70]. Free iron complexes with DOX and through the Fenton reaction create more ROS, thereby inducing ferroptosis. To go a step further, some scholars have focused their attention on mitochondria. They suggested that mitochondria are the main site of DOX-induced ferroptosis [71–73] and that targeting mitochondrial antioxidants can inhibit DIC [62]. DOX can cause mitochondrial iron overload by affecting mitochondrial ferritin [74], ABC protein-B8 [71, 75], frataxin [76], etc. and mitochondrial GPX4 is more important in antiferroptosis than cytoplasmic GPX4 [77].

### 3.7. Ca^2+^ Dysregulation

DOX can also induce DIC by affecting Ca^2+^ homeostasis in cardiomyocytes. DOX can increase the Ca^2+^ intake by increasing the current of L-type calcium channel, activate the RyR2 receptor to increase the Ca^2+^ release from sarcoplasmic reticulum (SR), inhibit SERCA2A to reduce the Ca^2+^ reuptake of SR, and activate CaMKII to cause SR Ca^2+^ leakage, which lead to the accumulation of intracellular Ca^2+^, thereby inducing DIC [78–82]. One study suggested that the degradation of titin caused by the accumulation of intracellular Ca^2+^ through the activation of calpain is an early event in DIC [83]. In addition, DOX-induced Ca^2+^ disturbance is closely related to ROS and apoptosis. Elevated ROS induced by DOX lead to intracellular Ca^2+^ overload. Accumulated Ca^2+^ results in nuclear translocation of NFAT by activating calcineurin, which activated Fas/FasL-mediated apoptosis, while DOX-induced apoptosis can be reversed by Ca^2+^ chelator and antioxidant [84]. Besides, increased Ca^2+^ also induced cardiomyocyte apoptosis by activating calcium-dependent CaMKII [85].

## 4. The Mechanism of EXOs against DIC

In recent years, it has been demonstrated that EXOs harbor a variety of miRNAs, lncRNAs, and proteins which may be transferred to cardiomyocytes through cell endocytosis or membrane fusion and modulate their function [10, 15, 86]. Different components contained in EXOs from different parent cells can inhibit DIC by acting on different signaling pathways. The mechanisms of EXOs against DIC are as follows:

### 4.1. Antiapoptosis

**Figure 3:**
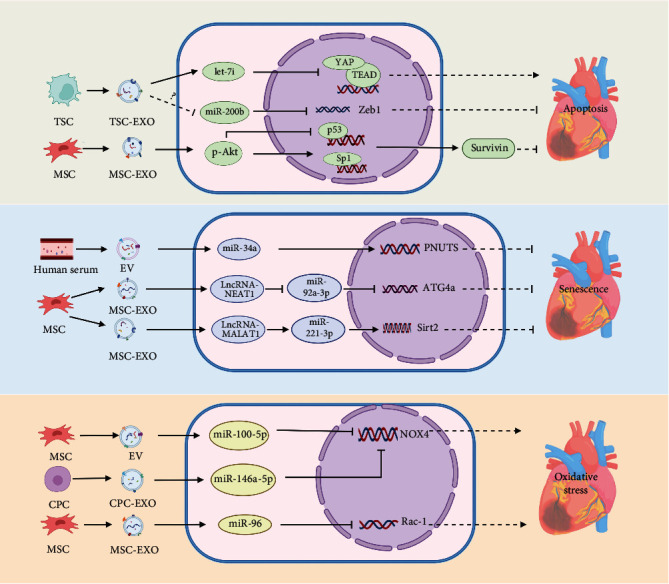
The mechanisms of EXOs against DIC involved in antiapoptosis, antisenescence, and antioxidative stress. TSC: trophoblast stem cell; TSC-EXO: exosome derived from trophoblast stem cell; MSC: mesenchymal stem cell; MSC-EXO: exosome derived from mesenchymal stem cell; YAP: yes associated protein; Zeb1: zinc finger E-box-binding protein 1; Sp1: specificity protein 1; EV: extracellular vesicle; PNUTS: phosphatase 1 nuclear targeting subunit; ATG4a: autophagy-related genes 4a; Sirt2: silent information regulator 2; CPC: cardiac progenitor cell; CPC-EXO: exosome derived from cardiac progenitor cell; NOX4: NADPH oxidase 4; Rac1: ras-related C3 botulinum toxin substrate 1.

Several studies have shown that EVs can alleviate DOX-induced cardiomyocyte apoptosis (Figure 3). Current studies mostly focus on EXO-loaded miRNAs. Generally speaking, EXOs acted as miRNA carriers, delivering miRNAs from parental cells to cardiomyocytes, altering the expression of their target genes and thus exerting antiapoptotic effects. EXOs derived from trophoblast stem cells (TSC-EXOs) are abundant in let-7i, which could exert antiapoptosis and antifibrosis effects in DOX-induced dilated cardiomyopathy (DCM) models both in vivo and in vitro by inhibiting the yes associated protein (YAP) signaling pathway. In let-7i mimic-treated cardiomyocytes group, apoptosis-related biomarkers Annexin V and cleaved caspase 3 were decreased, the expression of Bcl2 was increased, and related biomarkers (YAP1, CTGF, and TEAD1) in the YAP signaling pathway were inhibited, and myocardial fibrosis in pathological staining is mitigated [87]. Ni et al. demonstrated that TSC-EXOs protected the heart from DOX-induced apoptosis through the miR-200b/zinc finger E-box-binding protein 1 (Zeb-1) pathway. Specifically speaking, TSC-EXOs downregulated the expression of miR-200b in cardiomyocytes, thereby increasing the transcription of Zeb1. However, the mechanism by which TSC-EXOs downregulated miR-200b was still unclear, and they thought that it might be related to the lncRNAs in EXOs [88]. EVs, acting as miRNA carriers, can also carry miRNAs to target cells [89, 90]. MicroRNAs encapsulated in EVs are the important genetic material that drives cardiac repair. There was a study that demonstrated treatment with EVs derived from MSCs (MSC-EVs) before DOX treatment enhanced H9C2 cell viability. Mechanistically, miR-199a-3p in MSC-EVs upregulated p-Akt levels, thereby inhibiting the activation of transcription factor P53 and promoting the activation of Sp1, thus increasing the expression of antiapoptotic factor survivin to alleviate DIC [91]. One study showed that miR-100-5p in MSC-EVs exerted antiapoptotic effects in AC16 cells by inhibiting the expression of its target gene NOX4. This protective effect of MSC-EVs was reversed when MSC-EVs were transfected with miR-100-5p inhibitors or NOX4 was overexpressed [92].

### 4.2. Antisenescence

The mechanism by which EXOs alleviate DIC can be achieved by the antisenescence of cardiomyocytes, which is characterized by more cells escaping from the G0/G1 phase, the decreased expression of the cellular senescence-related genes p27, p53, p21, and p16, a lower percentage of SA-*β*-gal-positive cells, and the increase in telomere length and activity [93–95]. There was a study showed that it involved the EXOs/lncRNA–NEAT1/miR-221-3p/Sirt2 pathway. Abundant lncRNA–NEAT1 in EXOs derived from MSCs pretreated with macrophage migration inhibitory factor (MIF) (MSC-EXOs^MIF^) can improve the content of Sirt2 by targeting miR-221-3p, thus playing the antisenescence role. Moreover, siRNA-lncRNA-NEAT1 and miR-221-3p mimic transfection blocked the protective effect of MSC-EXOs^MIF^ [95]. Xia et al. suggested that EXOs secreted by MSCs pretreated with hypoxia (MSC-EXOs^hypoxia^) exerted antisenescence effects through the lncRNA-MALAT1/miR-92a-3p/ATG4a axis. MSC-EXOs^hypoxia^ transferred lncRNA-MALAT1 to cardiomyocytes, reducing the expression of targeted miR-92a-3p through ceRNA mechanisms, upregulating the expression of the ATG4a gene, thus exerting a rejuvenation effect. In addition, MSC-EXOs^hypoxia^ can also improve the mitochondrial metabolic disorder caused by DOX, which is manifested as the decrease of Fabp3, Fabp4, and Mtfp1 and the increase of Cox4i2, HSPa1a, and Atp1b2. After the lncRNA-MALAT1 knockdown, miR-92a-3p overexpression, or silencing of ATG4a, the above two protective mechanisms of MSC-EXOs^hypoxia^ were inhibited. Notably, cardiomyocytes without hypoxic preconditioning were slightly protective, and hypoxic preconditioning enhanced the cardioprotective effect of MSC-EXOs [93]. Besides, Liu et al.'s research suggested that human serum EVs exerted the antiaging effect in DIC H9C2 cells models. It was achieved by suppressing the expression of miR-34a and promoting the expression of its target gene phosphatase 1 nuclear targeting subunit (PNUTS). The miR-34a mimics and silence PNUTS can reverse the antiaging effect of EVs [94]. (Figure 3).

### 4.3. Antioxidative Stress

EXOs can combat DOX-induced oxidative stress (Figure 3). This was related to the antioxidant proteins and miRNAs contained in EXOs. Proteomics analysis revealed that EXOs from human right cardiac atrial appendage tissue contained more than 70 proteins involved in redox processes, especially SOD2, thrombospondin 1, and collagen 1A1 [96]. miR-96 in MSC-EXOs protected the heart from oxidative stress both in vivo and in vitro, and this effect was achieved through the inhibition of its target gene Rac-1. Compared with the EXOs+miR-96 inhibitor group and NC siRNA group, SOD and GSH-Px were increased, and malondialdehyde was decreased [97]. In addition, it was suggested that MSC-EVs inhibited DOX-induced oxidative stress in AC16 cells through the downregulation of NOX4 induced by miR-100-5p. When MSC-EVs were transfected with miR-100-5p inhibitors or NOX4 was overexpressed, the antioxidant effect was rescued [92].

### 4.4. Anti-inflammation and Antipyroptosis

There have been many studies showing that EXOs can improve the inflammatory microenvironment of cardiomyocytes and reduce the production of proinflammatory mediators (such as TNF-*α*, IL-1*β*, IL-1, and IL-6) [49, 87, 88, 96–99]. CPC-EXOs derived from human cardiac atrial appendage specimens were rich in miR-146a-5p and can enter cardiomyocytes, thereby inhibiting the transcription of their target genes Traf6 and Irak-1 and reducing the infiltration of CD68^+^ inflammatory cells [96]. In DOX-induced DCM mice models, after being injected with EVs derived from ECs overexpressing Krüppel-like factor 2 (EC-EVs^KLF2^), the levels of proinflammatory cytokines (IL-1*β* and TNF-*α*) were decreased, and the levels of anti-inflammatory cytokine IL-10 were increased, due to its ability to inhibit C-C chemokine receptor 2-mediated the migration of Ly6C^high^ Mo/Mø in bone marrow [99]. Besides, in the same mice models, MSC-EXOs could also alleviate the inflammatory environment. Mechanistically, MSC-EXOs decreased proinflammatory Ly6C^high^ monocytes and increased anti-inflammatory Ly6C^low^ macrophages by activating the transcription factor JAK2 and its downstream STAT6 [98]. In addition, a study showed that the anti-inflammatory effect of MSC-EXOs was related to their loaded miR-96, which inhibited DOX-induced activation of the Rac1 gene in cardiomyocytes, thereby reducing TNF-*α*, IL-6, and IL-1*β* [97].

**Figure 4:**
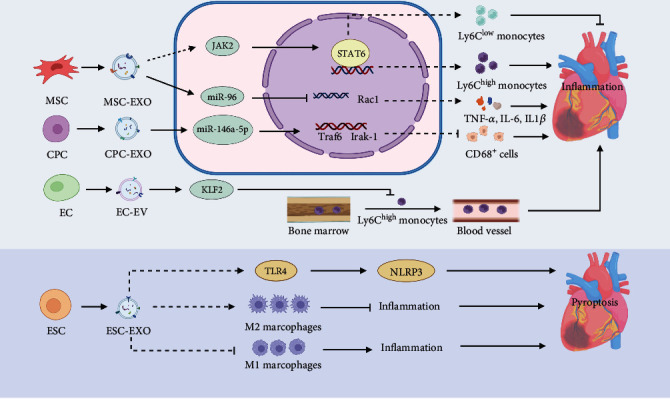
The mechanisms of EXOs against DIC involved in anti-inflammation, and antipyroptosis. MSC: mesenchymal stem cell; MSC-EXO: exosome derived from mesenchymal stem cell; CPC: cardiac progenitor cell; CPC-EXO: exosome derived from cardiac progenitor cell; EC: endothelial cell; EC-EV: extracellular vesicle derived from endothelial cell; JAK2: janus kinase 2; STAT6: signal transducer and activator of transcription 6; Rac1: ras-related C3 botulinum toxin substrate 1; TNF-*α*: tumor necrosis factor-alpha; IL-6: interleukin-6; IL-1*β*: interleukin-1*β*; ESC: embryonic stem cell; ESC-EXO: exosome derived from embryonic stem cell; TLR4: toll-like receptor-4; NLRP3: NLR family pyrin domain containing 3; KLF2: Krüppel-like factor 2.

As an inflammatory programmed cell death, pyroptosis is also involved in the occurrence and development of DIC. Researches were suggesting that embryonic stem cell-derived EXOs (ESC-EXOs) inhibited the activation of TLR4 and inhibit the formation of NLRP3, thereby improving the inflammatory environment and protecting cardiomyocytes from pyroptosis both in the DIC mice models and H9C2 cells models [49, 100]. These protective effects may be related to ESC-EXOs containing more anti-inflammatory cytokines (IL-4, IL-9, and IL-13) and fewer proinflammatory cytokines (TNF-*α*, TNFR1, IL-12, and Fas ligand) [49]. Singla et al. believed that the inflammatory microenvironment caused by DOX was related to the activation of the MAPK signaling pathway, and ESC-EXOs could inhibit the activation of these signaling pathway proteins (MyD88, p-P38, and p-JNK). In addition, the anti-inflammatory effect of ESC-EXOs may also be related to promoting the conversion of MI to M2 macrophages [100] (Figure 4).

The mechanisms underlying DIC are complicated; in addition to the above mechanism, autophagy, topoisomerase 2*β* inhibition, necroptosis, etc. are also involved [47, 101, 102]. However, unfortunately, there are no relevant studies on the protective effects of EXOs or EVs from DIC by regulating these mechanisms.

## 5. EXOs as Diagnostic Biomarkers for DIC

DIC can be divided into early-onset and late-onset. For early-onset, it is mostly reversible. Early detection and active intervention can prevent irreversible cardiac damage [4]. For delayed cardiotoxicity, if DIC is detected and intervened early, the patient is likely to have good functional recovery [103]. Strategies for screening and detection of cardiotoxicity include echocardiography, nuclear imaging, cardiac magnetic resonance, and biomarkers (troponin and natriuretic peptides) [4, 10]. The Cardio-Oncology Study Group of the Heart Failure Association and the Cardio-Oncology Council of the European Society of Cardiology recommend high-sensitivity troponin (hs-cTN) as a biomarker for early DIC because it can sensitively identify early myocardial damage caused by DOX and predict left ventricular dysfunction [104]. However, the specificity of hs-cTN in the diagnosis of DIC is not satisfactory, as it can also be elevated in other cases, such as hypertensive emergencies and renal failure [105, 106]. And the increase in troponin means that the cardiomyocytes have been damaged. Therefore, biomarkers, which are more sensitive and specific than troponin, need to be explored.

In recent years, EXOs have attracted the attention of many scholars as biomarkers for disease diagnosis. Firstly, EXOs are existed almost in all biological fluids and can be secreted by almost all cells, so it is theoretically possible to isolate EXOs from a patient's body fluids, such as serum or urine, for the diagnosis of disease. Secondly, the molecular characteristics of cargoes in EXOs reflect the phenotype of the cells from which they originated. Thirdly, the biomarkers in EXOs are more stable due to the encapsulation of the plasma membrane [19, 23, 107].

Table 1 shows potential EXOs or EVs cargoes that could be used as biomarkers for DIC. After DOX treatment, damaged myocardial tissue released EVs containing brain/heart glycogen phosphorylase (PYGB) into the blood. It can serve as a potential early biomarker of DIC that is more sensitive than cTnI, which could be detected in serum by proteomics. Yarana et al. and Zhu and Gius demonstrated significant differences in EVs-PYGB between saline and DOX-treated mice as early as 24 hours after treatment, whereas differences in cTnI were not detected until 72 hours after treatment in DIC mice models [107, 108]. Besides, in the DIC dog models, DOX caused changes in the expression profiles of miRNAs in circulating EXOs, as shown by miR107 and miR-146a which were significantly decreased, and the level of miR-502 was increased. Notably, during a total of 5 DOX treatments, the elevation of miR-502 appeared before the 3rd treatment, which was earlier than the changes in cTnI and echocardiographic parameters. Therefore, miR-502 in circulating EXOs can serve as a potential biomarker for DIC [109]. In addition, Li et al. thought that exosomal circ-SKA3 can be a candidate biomarker for DIC. After being exposed to 5 *μ*M DOX, human cardiomyocyte-like AC16 cells secrete EXOs enriched in circ-SKA3 internalized by recipient AC16 cells by docking and fusing to the cytomembrane, which can enhance cardiotoxicity via the miR-1303/TLR4 axis [110].

Components such as proteins, lipids, RNA, and miRNAs in EXOs may serve as diagnostic and harbingers of DIC. However, there are few related studies in this field at present, and we have only found a few articles. Moreover, there is a lack of relevant studies of serum EXOs in humans. In addition, it is unclear whether different tumor types and different underlying cardiac function states of patients affect the changes in the content of EXOs derived from cardiomyocytes in serum.

## 6. Prevention and Treatment of EXOs for DIC

The strategies of EXOs to alleviate DIC can be summarized into two major aspects: first, EXOs as nonimmunogenic nanosized vesicles (EXOs-DOX) improve the delivery efficiency of DOX and the uptake capacity of tumor cells to DOX, enhancing the anticancer effect of DOX, thereby reducing the dosage of DOX, indirectly reducing DIC; second, some contents in EXOs can directly act on the heart, inhibit cardiac damage caused by DOX, and directly treat DIC [10]. There are many studies on EXOs enhancing DOX's anticancer efficacy [24, 111–114], and the therapeutic part of this article focuses on the direct effect of the EXOs described above, as well as the direct evaluation of cardiotoxicity in the article on the role of EXOs as DOX delivery carriers (Table 2).

In acute DOX-induced DCM mice models, after being injected with human cardiac stem cell-derived EXOs (CSC-EXOs), the impaired heart function was improved, showing that both shortening fraction (FS) and ejection fraction (EF) were recovered. In addition, TUNEL staining showed that CSC-EXOs could inhibit cardiomyocyte apoptosis, Masson's trichrome staining revealed that CSC-EXOs could inhibit cardiomyocyte fibrosis, and H&E staining showed that CSC-EXOs could inhibit cardiac immune response [115].

miR-21a-5p was considered to be a potent cardiac protective miRNA in multiple studies. EXOs loaded with miR-21a-5p can be used to protect the heart from DOX damage [116–118]. However, the liver and spleen preferentially ingest EXOs and reduce their protective effects attributed to the mononuclear phagocyte system [114, 119]. Wan et al. demonstrated that clathrin played an important role in endocytosis by macrophages. Based on this, they invented a new two-step strategy to prevent DIC. Prior injection of EXOs^blocking^ (EXOs encapsulated with siClathrin) reduced the localization of EXOs in the liver and spleen while improved the cardiomyocyte localization, therefore strengthening the beneficial effect of EXOs^therapeutic^ (EXOs encapsulated with miR-21a-5p) in DIC model (5 mg/kg, IP; every week for 4 injections) which was characterized by higher EF and FS [120]. Martins-Marques et al. demonstrated that the gap junction protein connexin43 (Cx43) in EVs produced by HEK-293 cells was essential to reduce DIC because it formed a channel that accelerated the release of intravesical content into cardiomyocytes. Compared with EVs^Cx43-^-DOX treatment, EVs^Cx43+^-DOX increased the transverse diameter of cardiomyocytes in HE staining, decreased fibrosis in Sirius staining, and downregulated oxidative stress indicators COX2 and HSP25 [121]. Wei et al. believed that MSC-EXOs as DOX carriers enhanced cellular uptake efficiency of DOX in osteosarcoma MG63 cells, while its effect on H9C2 cardiomyocytes exhibited opposite effects which were manifested by higher cell viability and IC50 [122]. One research has shown that DOX in EXOs originated from MDA-MB-231 and HCT-116 cell lines have a lower ability to cross human myocardial ECs than free DOX, which resulted in less DOX accumulation in the heart significantly [123, 124].

To further enhance the anticancer effect of EXOs-DOX, some medical technologies have been applied to EXOs. miR-21 in EXOs alleviated DIC [120, 125]. Sun et al. isolated EXOs from mouse plasma by centrifugation and then loaded miR-21 into EXOs by electroporation to alleviate DIC. By using ultrasound targeted microbubble destruction (UTMD), the delivery efficiency of miR-21 in EXOs to the heart was significantly enhanced, as manifested by the increased EF and E/A ratio [126]. Li et al. demonstrated EXOs coated with surface-carboxyl superparamagnetic iron oxide nanoparticles (US) with A33 antibodies (A33Ab-US), also known as A33Ab-US-EXOs-DOX, enhanced the targeting ability of DOX to A33-positive colorectal cancer cells. Compared with free DOX and EXOs-DOX, A33Ab-US-EXOs-DOX was more effective against cancer both in vivo and in vitro, manifested by higher cell uptake rates, lower cell viability, lower half-maximal inhibitory concentrations, more necrosis and apoptosis of cancer cells, and smaller tumor volumes. More importantly, H&E staining showed no significant cardiotoxicity [127]. Quah and O'Neill transfected immature dendritic cells which lacked immunostimulatory markers on their surface (such as CD40, CD86, MHC-I, and MHC-II) [128] via iRGD-lamp2b to obtain EXOs containing lamp2b, which loaded DOX (iRGD-EXOs-DOX) targeted metastatic breast cancer tissues, achieving more precise and effective anticancer effects both in vivo and in vitro. Besides, iRGD-EXOs-DOX had fewer cardiac side effects than free DOX and empty EXOs loaded with DOX, manifesting as lower levels of CK-MB and AST, and H&E staining showed no significant pathological damage [129].

As nanoscale biofilm structures, therapeutic EXOs can be produced by patients themselves without immune responses. In addition, the special structure of EXOs enables them to be engineered to be loaded with therapeutic miRNAs, proteins, or DOX, making them an ideal drug carrier for the prevention and treatment of DIC. In addition, despite the great promise of EXOs for the treatment of DIC, there are some challenges. Different cell-derived EXOs have different characteristics and functions, and the relationship between EXOs' subsets and DIC efficacy needs to be further explored. In addition, the efficient isolation of EXOs, the standardization of EXO preparations, and the determination of the effective dose of therapeutic EXOs are also key issues that need to be solved urgently in the process of clinical transformation of EXOs [10, 18, 19].

## 7. Conclusions and Perspectives

In conclusion, this review highlights and summarizes current studies regarding the role of EXOs in DIC, with a focus on cardioprotection. Different cell-derived EXOs can inhibit DOX-induced oxidative stress, inflammation, senescence, apoptosis, and pyroptosis. Besides, some components in EXOs play an important role in the occurrence and development of DIC. By detecting these components, DIC can be diagnosed sensitively and specifically; that is, EXOs have the potential to serve as DIC biomarkers. As a natural low-immunogenic DOX delivery carrier, EXOs can improve the loading rate of DOX, increase the uptake rate of DOX by tumor cells, exert a stronger antitumor effect, and reduce the dosage of DOX, thus indirectly reducing DIC. In addition, EXOs-DOX can reduce the uptake of DOX by cardiomyocytes and the distribution of DOX in cardiomyocytes, thereby directly reducing cardiotoxicity. It is conceivable that EXOs hold the excellent prospect for diagnosis and treatment soon with further research.

## Figures and Tables

**Table 1:** Summary of studies using EXOs/EVs as biomarkers for DIC diagnosis.

EVs type	Component	Parts of EXOs/EVs	Study model	DOX administration	Distribution	Ref
EXOs	circ-SKA3	circRNA	Human cardiomyocyte-like AC16 cells	5 *μ*M for 24 h	—	[110]
EVs	PYGB	Protein	Male C57BL/6J mice	20 mg/kg, IP; a single dose	Serum	[107]
EXOs	miR-502	miRNA	Dogs diagnosed with sarcoma	30 mg/m^2^ for dogs > 15 kg and 1 mg/kg for dogs < 15 kg, IV; 2 to 3 weeks for 5 injections	Serum	[109]

EXOs: exosomes; EVs: extracellular vesicles; PYGB: brain/heart glycogen phosphorylase; IP: intraperitoneal injection; IV: intravenous injection.

**Table 2:** Summary of studies using EXOs/EVs for the treatment of DIC.

Parent cell	EVs type	Cargo	Cargo formulation	Study model	Antitumor parameters	Cardiotoxicity parameters	Ref
CSCs	EXOs	—	EXOs	In vivo (mice); in vitro (NRCMs)	NA	EF↑, FS↑, apoptosis↓, fibrosis↓, immune response↓	[115]
MSCs	EXOs	DOX	EXOs-DOX	In vivo (MG63 cells, H9C2 cells)	MG63 cells: cell uptake rate↑, cell viability↓	H9C2 cells: cell viability↑	[122]
MDA-MB-231, STOSE, MDAMB-231 CD63-GFP and STOSE CD63-GFP cell lines	EXOs	DOX	EXOs-DOX	In vivo (mice); in vitro (human myocardial endothelia cells)	Maximum tolerated dose of DOX↑, tumor volume↓	The ability to cross a reconstructed myocardial endothelial monolayer↓, vacuoles↓, and myofibril disorganization↓ in H&E staining	[124]
MDA-MB-231 and HCT-116 cell lines	EXOs	DOX	EXOs-DOX	In vivo (mice); in vitro (MDA-MB-231 cells)	Cell viability↓, tumor volume↓	Distribution of DOX in the heart↓, no cardiac damage in H&E staining	[123]
HEK-293 cells	EXOs	miR-21a	EXOs^siClathrin^, followed by EXOs^miR-21a^	In vivo (mice)	NA	miR-21a-5p expression in heart↑, EF↑, FS↑	[120]
HEK-293 cells	EVs	Cx43, DOX	EVs^Cx43+^-DOX	In vivo (mice); in vitro (4T1^luc2^ cells)	Cell viability↓, cell proliferation↓, cell motility↓, colony formation↓, tumor growth↓, apoptosis↑	Fibrosis↓, histopathological changes↓, COX-2↓, HSP25↓	[121]
NA	EXOs	miR-21	UTMD+EXOs^miR-21^	In vivo (mice)	NA	miR-21 delivery efficiency↑, EF↑, E/A value↑	[126]
LIM1215 cells	EXOs	DOX	A33Ab-US- EXOs-DOX	In vivo (mice); in vitro (LIM1215 cells)	Cell uptake rate↑, cell viability↓, half-maximal inhibitory concentrations↓, necrosis↑, apoptosis↑, tumor volume↓	Apoptosis↓, cardiac damage↓	[127]
Mouse immature dendritic cells	EXOs	DOX	iRGD-EXOs-DOX	In vitro (MDA-MB-231 cells); in vivo (mice)	DOX delivery efficiency↑, cell viability↓, tumor volume↓	CK-MB↓, AST↓, no cardiac damage in H&E staining	[129]

CSCs: cardiac stem cells; EXOs: exosomes; NRCMs: neonatal rat cardiomyocytes; MSCs: mesenchymal stem cells; Cx43: the gap junction protein connexin43; UTMD: ultrasound-targeted microbubble destruction; FS: shortening fraction; EF: ejection fraction; COX-2: cyclooxygenas-2; HSP25: heat shock protein 25; CK-MB: creatine kinase-MB; AST: aspartate transaminase; A33Ab-US-EXOs-DOX: DOX loaded in exosomes coated surface-carboxyl superparamagnetic iron oxide nanoparticles with A33 antibodies.
